# How to Set Up Genetic Counselling for Inherited Macular Dystrophies: Focus on Genetic Characterization

**DOI:** 10.3390/ijms24119722

**Published:** 2023-06-03

**Authors:** Raffaele Raimondi, Fabiana D’Esposito, Tania Sorrentino, Panos Tsoutsanis, Francesco Paolo De Rosa, Elisa Stradiotto, Gianmaria Barone, Angelica Rizzato, Davide Allegrini, Ciro Costagliola, Mario R. Romano

**Affiliations:** 1 Department of Biomedical Sciences, Humanitas University, Via Rita Levi Montalcini 4, Pieve Emanuele, 20072 Milan, Italy; 2 Imperial College Ophthalmic Research Group (ICORG) Unit, Imperial College, 153-173 Marylebone Rd, London NW1 5QH, UK; 3 Department of Neurosciences, Reproductive Sciences and Dentistry, University of Naples Federico II, Via Pansini 5, 80131 Napoli, Italy; ciro.costagliola@unina.it; 4 Ospedale Regionale di Lugano, 6900 Lugano, Switzerland; 5 Eye Center, Humanitas Gavazzeni-Castelli, 24125 Bergamo, Italy

**Keywords:** macular dystrophies, inherited retinal dystrophies, genetics, genetic testing, genotype/phenotype correlation

## Abstract

Inherited macular dystrophies refer to a group of degenerative conditions that predominantly affect the macula in the spectrum of inherited retinal dystrophies. Recent trends indicate a clear need for genetic assessment services in tertiary referral hospitals. However, establishing such a service can be a complex task due to the diverse skills required and multiple professionals involved. This review aims to provide comprehensive guidelines to enhance the genetic characterization of patients and improve counselling efficacy by combining updated literature with our own experiences. Through this review, we hope to contribute to the establishment of state-of-the-art genetic counselling services for inherited macular dystrophies.

## 1. Introduction

In the wide and heterogeneous spectrum of inherited retinal dystrophies (IRDs), inherited macular dystrophies (IMD) encompass a group of degenerative pathologies primarily affecting the macula with anomalies involving the retinal pigmented epithelium (RPE) and the sensory retina. Progressive macular atrophy, which is usually bilateral and fairly symmetrical, causes significant visual loss, beginning with impairment of the central visual function [1,2]. Affected individuals must rely on their peripheral visual fields to perform their normal visual functions, including those for which peripheral retina would not normally be designated, such as fine discrimination of images. In order to achieve this, patients adopt certain retinal areas adjacent to the macula to be used to fixate objects, just as the fovea would normally. These areas are referred to as preferred retinal loci (PRL) [3]. Somewhat frequently, dystrophies are not purely macular; they can also involve the mid-peripheral and peripheral retina, either at the same time as the central involvement or subsequently.

### 1.1. Most Common Types of IMDs

**Figure 1:**
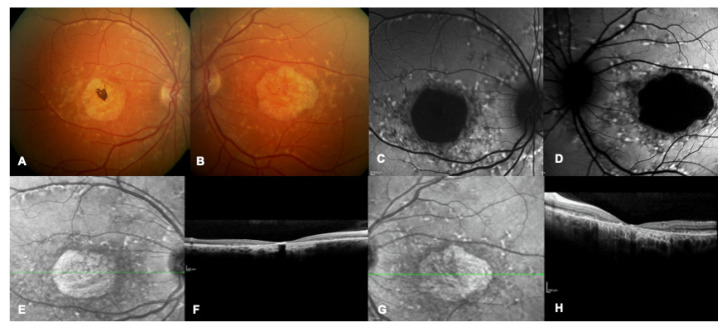
Multimodal imaging of a patient with a confirmed genetic diagnosis of ABCA4-related macular dystrophy. (**A**,**B**) Bilateral multicolor fundus images showing yellow flecks and advanced macular atrophy. (**C**,**D**) Bilateral blue autofluorescence showing macular hypoautofluorescence consistent with an advanced stage of the disease. (**E**–**H**) Bilateral infrared images show diffused flecks and foveal atrophy and bilateral OCT highlights loss of outer retinal layers and absence of foveal layers.

The most common IMD is Stargardt disease, with a worldwide prevalence of 1:8000 to 1:10,000; onset is often in the first or second decade, but, not unusually, onset can occur later (Figure 1). Patients experience a progressive decay of visual acuity, coupled with dyschromatopsia and photophobia. It is characterized by RPE changes at the level of the macula, gradually progressing towards atrophy of the RPE/photoreceptors complex, and in most cases by the presence of retinal flecks. Flecks are yellow-white lesions due to an excessive storage of lipofuscin, resembling drusen, but less rounded and regular in shape and usually larger. They are predominantly located around the macula with variable midperipheral distribution and their presence and/or distribution varies over time [1]. A typical aspect of Stargardt disease is peripapillary sparing of the RPE, very evident at fundus autofluorescence (FAF) imaging. Although gradually substituted by FAF, fluorescein angiography displays a typical sign, the “dark choroid”, in about 80% of patients. At the end stage of the disease, the outer layer of the retina is lost, while the pigmented epithelium is degenerated, resulting in the fusion of gliotic retina with the Bruch’s membrane.

Electrodiagnostic tests (EDTs) usually display an abnormal Pattern and Multifocal Electroretinogram (PERG and mfERG). Lois et al. [4] described three subtypes of *ABCA4*-related ERG alterations: 1. PERG abnormalities with normal scotopic and full-field ERG, in disease confined to the macula; 2. Additional full-field photopic ERG abnormalities, reflecting generalized cone dysfunction; 3. Abnormalities in the scotopic ERG reflecting an additional rod dysfunction. These differences in the ERG patterns unsurprisingly reflect phenotypic heterogeneity of *ABCA4*-related IRDs.

Most cases of Stargardt disease are autosomal recessive and are caused by bi-allelic pathogenic variants in the *ABCA4* gene (OMIM# 601691), a large 50 exons gene, encoding for a protein which is part of an ATP-binding cassette family that is responsible for transporting end products of cellular metabolism out of the photoreceptors. Impairment of ABCA4 protein causes the accumulation of end products, that is potentially toxic. Although over 2300 variants have been reported in the literature [5], in a significant proportion of patients, a second variant, necessary to confirm compound heterozygosity in this recessive condition, cannot be identified. The reason could be that either some variants can be in “hidden” intronic or regulatory regions of the gene, or that hypomorphic variants are incorrectly classified as benign, or even that the disease-causing gene is a different one [6]. Evidence has shown that age of onset is related to severity of *ABCA4* pathogenic variants. Childhood onset disease is often associated with more severe variants, such as nonsense type, whereas adult-onset disease with less deleterious variants, such as missense mutations, causing a milder disease phenotype [1,3]. Being a recessive condition, the combination of types of the two pathogenic variants influences the phenotype severity [7].

Currently 43 clinical trials for *ABCA4*-related retinal dystrophies are registered; of these, 28 are interventional (https://clinicaltrials.gov, accession 8 May 2023).

Stargardt-like phenotypes can be caused by different genes. The most common are the dominant genes *ELOVL4*, *PROM1*, or *PRPH2*. *RDH12*, a recessive gene causing Leber Congenital Amaurosis, has been described as possibly displaying a Stargardt-like phenotype, essentially in the peripapillary sparing at autofluorescence and early macular involvement, but with a full-field abnormal ERG [8]. A recent paper [9] described Stargardt-like phenotypes caused by *RIMS1*, *CRX*, *CRB1*, and *RDH12* genes (Table 1).

**Figure 2:**
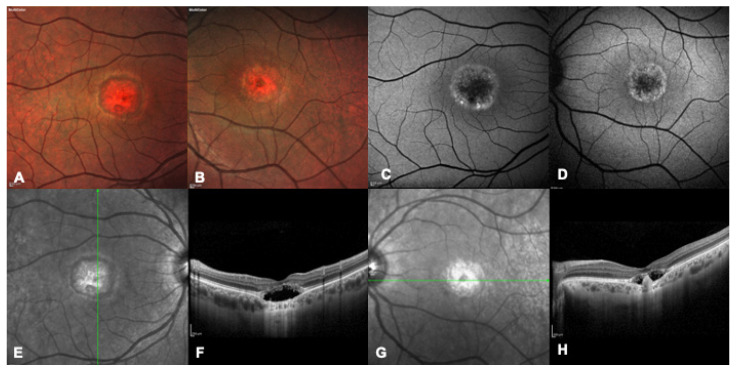
Multimodal imaging of a 32 years-old patient with a confirmed genetic diagnosis of BEST1-related macular dystrophy. (**A**,**B**) Bilateral multicolor fundus pictures showing bilateral advanced foveal atrophy. (**C**,**D**) Bilateral blue autofluorescence image showing marked foveal hypofluorescence surrounded by a ring of hyper autofluorescence. (**E**,**F**) Right eye infrared and OCT image showing hyporeflective subretinal fluid and a thickened interdigitation zone, consistent with the pseudohypopyon stage. (**G**,**H**) Left eye infrared and OCT image showing hyporeflective subretinal fluid and a central fibrotic scar, consistent with the vitelliruptive stage.

**Figure 3:**
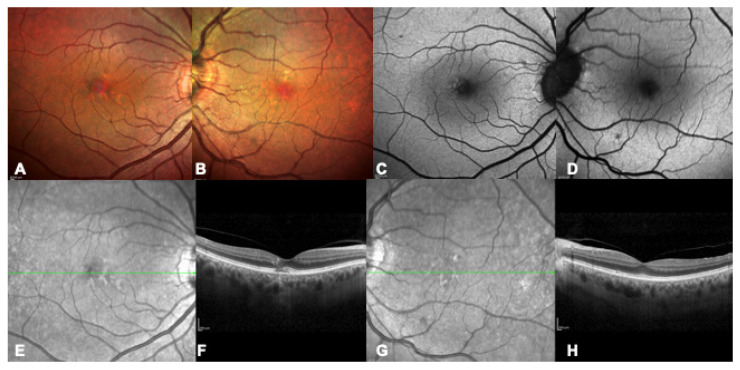
Multimodal imaging of a 73 years-old patient with a confirmed genetic diagnosis of *BEST1*-related macular dystrophy, father of the patient in Figure 2, as a clear example of interfamiliar variability. (**A**,**B**) Bilateral multicolor fundus pictures showing scattered speckle lesions. (**C**,**D**) Bilateral blue autofluorescence image showing initial hyper-autofluorescent lesions. (**E**–**H**) Bilateral infrared and OCT images with early alterations including disruption of the photoreceptors layers (**F**).

The second most common IMD is Best disease, with a prevalence estimated at 1:5500 in a North American population but varying in different populations [10] (Figure 2 and Figure 3). Best disease is an autosomal dominant condition related to pathogenic variants in the *BEST1* gene; this gene encodes for the bestrophin protein, an anion channel and a regulator of intracellular calcium signaling [11]. Although different classifications have been proposed, Best disease essentially evolutes through six stages, based on changes in the appearance of the macula and associated changes in vision. In the earliest stage, known as Stage I or Previtelliform, vision is normal and only subtle changes can be noted in the retinal pigment epithelium (RPE), with a central tiny honeycomb structure and abnormal electrooculogram (EOG). Stage II, or Vitelliform, is characterized by the classic “egg-yolk” lesion with normal or mildly decreased vision. Around 30% of patients with Stage II also have ectopic lesions. In Stage III or Pseudohypopyon, there is a layering of vitelliform material in the inferior part of the lesion, and vision is similar to Stage II. Stage IV, or Vitelleruptive, is characterized by the breakup of the material that gives a “scrambled egg” appearance, and vision may be similar or mildly decreased when compared to Stage I/II. In Stage V, or Atrophic, there is central RPE and retinal atrophy, and vision may range from 20/30 to 20/200. Finally, in Stage VI, about 20% of patients develop a choroidal neovascularization (CNV) [12]. As evident in Figure 2 and Figure 3, representing a daughter with a more severe phenotype than her father, intrafamilial variability is very common. Patients can have a later onset of the condition, which is defined as adult-onset Vitelliform Macular Dystrophy (AVMD) and is linked to *PRPH2* gene. EOG will be normal in these cases, while pattern or multifocal ERGs will support the diagnosis (Table 1).

**Figure 4:**
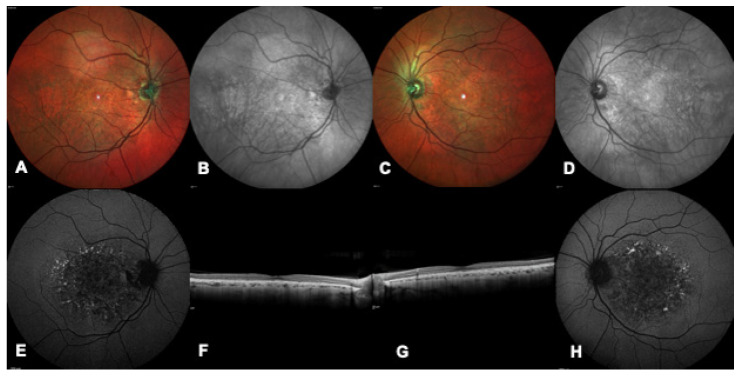
Multimodal imaging of a patient with a confirmed genetic diagnosis of *PRPH2*-related macular dystrophy. (**A**,**C**) Bilateral multicolor images show a bilateral white foveal lesion more prominent in the left eye (**C**). (**B**,**D**) Bilateral infrared images highlight a bilateral foveal and round shaped lesion. (**E**,**H**) Bilateral blue autofluorescence showing decreased autofluorescence in the foveal area and speckled hyper-autofluorescent lesions in the peri-macular area. (**F**,**G**) Bilateral macular OCT demonstrating rarefaction of the ellipsoid zone.

The *PRPH2* gene (OMIM # 179605, consisting of three exons) encodes for a structural protein located in the rim portion of rod and cone outer segment discs. It is inherited dominantly, with the exception of a digenic form of retinitis pigmentosa (RP) coupled with *ROM1* variants. To date, over 340 variants have been described as related to a wide spectrum of IRDs, from macular dystrophies (Figure 4) to cone and cone-rod dystrophies and retinitis pigmentosa, sometimes even with an intra-familial variability both in terms of severity and phenotype. Macular phenotypes can have the characteristics of butterfly-shaped pattern dystrophy, AVMD, Stargardt-like macular dystrophy, and/or central areolar choroidal dystrophy. Not uncommonly, rods photoreceptors dysfunction becomes evident in later stages of an initially exclusively macular alteration [13].

X-linked retinoschisis (XLRS) is a form of juvenile-onset retinal degeneration occurring in males, with a prevalence of 1:15,000 to 1:30,000. The age of onset is typically within the first decade of life. The characteristic feature of this trait is represented by bilateral, fairly symmetric, macular schises that are seen at fundoscopy as small superficial cysts arranged in a stellate pattern radiating from the fovea (classic spoke-wheel appearance). These cysts collapse with time, causing non-specific macular atrophic changes. Approximately 50% of patients show some grade of peripheral fundus anomalies [14]. X-linked retinoschisis is caused by pathogenic variants in the *RS1* gene (OMIM #300839, consisting of six exons) that encodes for retinoschisin, a cell surface protein found in photoreceptors as well as bipolar cells [1,15]. Retinoschisin directs protein translocation to the exterior of the cell and has an adhesive function that helps to maintain the structure of retinal layers and establish appropriate synaptic connections [16]. About 450 different variants have been described. Visual acuity can vary between 20/25 and 20/400 or worse. Electronegative electroretinogram (ERG) in response to a bright flash under dark adapted condition and a reduced b/a ratio in response to a single flash under photopic condition are typical of this disease (Table 1).

North Carolina Macular Dystrophy is a rare autosomal dominant form, displaying a variable phenotype, but is essentially characterized by small drusen-like deposits (grade 1), then becoming confluent (grade 2), eventually progressing towards a large oval area of macular atrophy (grade 3). Visual acuity is usually preserved in grades 1 and 2 and full-field ERG remains normal. Genetically, MCDR1 type is caused by variants in the noncoding region of the DNase I hypersensitivity site *DHS6S1* (OMIM # 616842), which is thought to be a regulatory element of the retinal transcription factor gene *PRDM13* [17,18].

Dominant Drusen (or Malattia Leventinese, or Doyne Honeycomb Macular Dystrophy) is an autosomal dominant condition related to the presence of the only described related variant (p.Arg345Trp) in the *EFEMP1* gene (OMIM #601548, consisting of 12 exons). Patients remain typically asymptomatic until the fourth or the fifth decade, following which the main symptoms are metamorphopsias and visual acuity reduction. Basal laminar drusen displays a characteristic distribution over the posterior pole in typical radial pattern and around the edge of the optic nerve. Over time they increase in number and size and display a honeycomb pattern [19]. Choroidal neovascularization is a possible compliance of this condition, resembling an early onset of age-related macular dystrophy (AMD) [20].

Sorsby Fundus Dystrophy is an autosomal dominant retinal degeneration related to the presence of pathogenic variants in the *TIMP3* gene (OMIM #188826, consisting of five exons, with 39 variants reported). Visual acuity decay and metamorphopsias usually occur around the fourth to sixth decade, and choroidal neovascularizations are not uncommon [21].

Occult Macular Dystrophy (OMD) is characterized by normal fundus appearance and progressive loss of visual acuity. The known underlying gene is dominantly inherited *RP1L1* (OMIM #608581, four exons, 545 reported variants); this is also potentially a cause of RP when recessively inherited [22,23].

The *CDH3* gene (OMIM # 114021) causes a recessive form of macular dystrophy associated with hypotrichosis [24,25].

In the complex scenario of IRDs, some genes display a marked phenotypic heterogeneity, potentially causing either distinct macular dystrophies or diffused diseases. In particular, this is the case for genes *PROM1*, *IMPG1*, *FSCN2*, *OTX2*, *CRX*, *RAB28*, and *GUCA1A* [26,27].

When encountering phenotypes of macular dystrophies, differential diagnosis must be considered. This is especially the case with infectious diseases, such as toxoplasmosis (significantly resembling North Carolina MD), CMV, or toxocariasis. In addition, some systemic drugs with retinal toxicity may mimic the phenotype of an IRD, such as hydroxychloroquine, tamoxifen, and Pentosan Polysulfate Sodium [28]. These causes of similar phenotypes could be very misleading when seeking correct diagnosis and management, hence the importance of careful interviewing of the patient with ad hoc questions.

### 1.2. Transmission

All modes of genetic transmission can be recognized in IMDs: autosomal dominant, autosomal recessive, X-linked, and mitochondrial. A considerable proportion of patients, though, are defined as “sporadic” or “simplex” due to an unremarkable family history for the condition. In dominant pedigrees, the disease is inherited from an affected parent, who has a 50% risk of transmission. Male-to-male transmission is exclusively identifiable in this form. Not uncommonly, dominant genes display a very variable intrafamilial expression (particularly *PRPH2* and *BEST1*), leading to the pathologic trait identification in the transmitting parent following the proband’s genetic characterization.

In recessive genes, heterozygous carrier unaffected parents have a 25% risk of having an affected child. This form of transmission is characteristic of consanguineous families, where the carriers’ rate is considerably higher. In addition to known consanguinity, the possibility of a common origin from a so called “genetic isolate” should be investigated where a shared genetic background is more likely to be present for geographic, cultural, or religious reasons. In some families or communities with a high rate of consanguinity, recessive pedigrees may resemble dominant types due to the presence of affected individuals in subsequent generations.

X-linked pedigrees typically display the presence of affected males descending from carrier females. Carrier females can frequently show signs of the disease in variable grades, especially in the dominant x-linked forms, where one mutated allele is enough to cause the condition. In addition to the definition of dominant and recessive in X-linked traits, other mechanisms, such as skewed X-inactivation, should be considered. A carrier female would have a 50% risk of having an affected male or a carrier female child, while an affected male will have unaffected male and obligate carrier female offspring.

In mitochondrial inheritance, affected mitochondria are classically transmitted from the maternal ovum cytoplasm in a variable proportion (heteroplasmy), leading to variable expression of related conditions.

Sporadic patients can be affected by a condition following any of the described patterns of transmission. In most cases a recessive gene is identified where parents were not aware of being carriers. The same can happen in X-linked conditions or mitochondrial, but usually a family history is positive in these cases. A dominant variant can be present, either as a de novo mutation or in pedigrees with variable expression of the disease and clinically undiagnosed affected patients.

IMDs, and generally IRDs, are extremely heterogeneous, both genetically and phenotypically [29]. The same defective gene can be associated with multiple phenotypically different forms of dystrophy; the same phenotype can be caused by different genes and combinations of variants with different degrees of severity can cause different phenotypes. An extremely high level of variable inter- and even intra-familial expressivity is noted, leading to the conclusion that the effect of a specific genetic variant can be modulated by other genetic and/or epigenetic factors. Epigenetic modifications consist of phenotype modifications resulting from changes in proteins expression without alterations in the DNA sequence. These can include DNA methylation, chromatin remodeling, and RNA-binding proteins and miRNA [30].

Current trends indicate a clear need for a genetic assessment service in every tertiary referral hospital. In this review we summarize updated literature and merge it with our experience in order to provide a state of art guideline to improve genetic characterization of patients and counselling efficacy.

The peer-reviewed literature was analyzed and all relevant articles were selected. A literature search was conducted in October 2022 using Medline, the Cochrane Library, and databases of clinical trials; the searches were limited to studies published in English. The search strategy used the following MeSH terms and text words: genetic ophthalmology consult, hereditary macular dystrophy, and ophthalmology genetic therapy. The initial search yielded 276 citations. Abstracts of meeting presentations were not included in the analysis because of their limited data. The authors reviewed 205 abstracts and selected 134 articles of possible relevance to review in the full text. Only papers providing information used in the present work have been cited.

**Table 1:** Principal characteristics and therapeutic options for common inherited macular dystrophies.

IMD	Clinical Presentation	Imaging Presentation	Therapeutic Options	Ongoing Trials	Ref.
Stargardt and Stargardt-like disease	Decreased central vision FO: foveal atrophy, bull’s eye pattern, yellow macular pisciform flecks (fundus flavimaculatus)	OCT: photoreceptor layer and RPE atrophy OCT-A: choriocapillaris anomalies FAG: paramacular hyperfluorescence associated with flecks (bull’s-eye aspect) and dark (or ‘silent’) choroid (screen effect on normal choroidal fluorescence) FAF: elevated background autofluorescence, central macular hypoautofluorescence, hyperautofluorescent flecks interleaved with hypoautofluorescent areas, peripapillary sparing of the RPE changes.	Patients with Stargardt disease should avoid supplementation of vitamin A and exposition to bright light.	Drug therapies to reduce lipofuscin accumulation, gene therapies, and stem cell treatments. A recent trial explored the use of a lentivirus, equine infectious anaemia virus (EIAV) to carry the *ABCA4* gene.	[4,6,9,26,28,31,32,33,34,35,36]
Best disease/Adult Vitelliform Macular Dystrophy	Decreased vision, hyperopia (*BEST1*) FO: Egg yolk-like lesion (stage I-II), evolving to pseudohypopyon (stage III), reabsorption (stage IV) and geographic atrophy (stage V)	OCT: hyperreflective vitelliform lesion between the EZ and the RPE, cysts in the neurosensory retina FAF: hyperautofluorescent zones due to the lipofuscin accumulation, hypoautofluorescent zones due to the RPE atrophy	No therapeutic options are available. Choroidal neovascularization (CNV) can be treated with antiVEGF intravitreal therapy.		[11,37]
*PRPH2* (more common), *CTNNA1*	Often asymptomatic, patients may develop metamorphopsia, a slight decrease in vision and a delayed recovery from exposure to bright light. Rarely, patients may present with rapid vision loss due to development of CNV. FO: various pattern of lipofuscin accumulation due to RPE defects. Often intra-familial variability	OCT: subretinal hyperreflective lesions FAF: areas of hyperfluorescence or hypofluorescence corresponding to lipofuscin contents in the RPE	No therapeutic options are available, CNV can be treated with antiVEGF therapy.		[13,38]
X-linked retinoschisis	FO: Small superficial cysts arranged in a stellate pattern radiating from the fovea, evolving to non-specific atrophy in late stages. The peripheral retina may show RPE alterations and schisis	OCT: inner retinal layer schisis FAF: modification of normal foveal autofluorescence with a radial pattern	Oral acetazolamide or topic inhibitors of carbonic anhydrase (CAIs) may reduce cystic spaces, vitreoretinal surgery in patients who develop vitreous hemorrhage or rhegmatogenous retinal detachment	Ongoing trials about *RS1* gene therapy that demonstrated a good profile of security in animal models	[14,15,39,40,41]
North-Carolina macular dystrophy	Normal visual acuity in grade 1 and grade 2, central visual loss in grade 3.	FO: drusen (grade1), macular yellowish-white atrophic lesions (grade 2), colobomatous macular defects (grade 3)	No therapeutic options are avaliable		[17,18]
Doyne macular dystrophy	Asymptomatic until the 4th or the 5th decade, metamorphopsis and visual acuity reduction.	OCT: hyperreflective deposits between the RPE and Bruch’s membrane start as focal dome-shaped, saw-tooth, or diffuse elevations. As time passes, they tend to merge and become more confluent.	No therapeutic options are available. Choroidal neovascularization (CNV) can be treated with antiVEGF intravitreal therapy.		[20]
Sorsby fundus dystrophy	Around the ages of 40 to 60, decline in visual acuity and onset of metamorphopsias.	OCT: diffused drusenoid deposits, possible reticular pseudodrusen	No therapeutic options are available. Choroidal neovascularization (CNV) can be treated with antiVEGF intravitreal therapy.		[21]

## 2. Pattern of Care

### 2.1. Interviewing—Data Collection about Age at Onset, Symptoms, and Evolution

During a consultation for a genetic condition, patient history plays a pivotal role. Symptoms and age at onset should be investigated in depth. Although IMDs have highly heterogeneous clinical features, they do share some characteristics. These pathologies predominantly affect central vision, with some of them having associations with visible alterations in the peripheral retina (e.g., X-linked retinoschisis, *PRPH2* related dystrophies) [38].

The predominant symptom is visual acuity loss. In most dystrophies there is only a slight vision loss at an early stage that may become detrimental in late stages, almost invariably due to a process of retinal atrophy at the macular level, possibly leading to a central scotoma. Other possible symptoms observed in IMDs are metamorphopsias, impaired color vision, and photophobia. In advanced stages of disease, when mid peripheral and peripheral retina can be involved as well, patients can notice varying degrees of night blindness and difficulties in dark adaptation [42]. Choroidal neovascularization can complicate the phenotype, and occurs more frequently in some conditions, including *EFEMP1*, *TIMP3*, *BEST1*, and *PRPH2*-related dystrophies.

Age of symptoms’ onset is highly variable. Best VMD and Stargardt disease, the two most common macular dystrophies, show a bimodal and a trimodal peak of distribution in age of clinical onset, respectively; X-linked retinoschisis and developmental macular dystrophies such as North-Carolina MD typically arise during childhood, whereas patients affected by pattern dystrophies or dominant drusen tend to remain asymptomatic until the fifth decade or even throughout life [12].

Family history should be carefully recorded, and in case of conditions with variable expressivity, deep phenotyping should be undertaken for family members before classifying them as affected or unaffected (Table 2).

During first approach interviewing, special care should be taken to record any extraocular condition in order to recognize syndromic conditions, such as Psudoxanthoma Elasticum, Bardet-Biedl Syndrome, Juvenile Neuronal Ceroid Lipofuscinoses, or Mitochondrial Retinopathies.

The support of a clinical geneticist is crucial, especially for addressing co-morbidities investigations in syndromic cases and to address reproductive issues, such as risk of recurrence.

### 2.2. Phenotyping

Most macular dystrophies are characterized by typical and recognizable features seen during fundus ophthalmoscopy. Macular fundus abnormalities often precede the onset of symptoms and thus diagnosis is sometimes made incidentally during routine fundoscopy. Instead, in other cases, symptoms appear when no fundoscopic signs are visible and therefore the diagnosis could be delayed (e.g., early onset Stargardt, Occult Macular Dystrophy). Deep phenotyping is crucial for the subsequent genotype/phenotype correlation [27].

#### 2.2.1. Imaging

Fundoscopy is a critical step in diagnosing macular dystrophies; however, as mentioned, in some cases the clinical examination is not sufficient for the evaluation of the patient’s condition. The advent of sophisticated imaging has revolutionized the understanding of macular structural changes that occur in macular dystrophies, and it is crucial in diagnosing and monitoring progression of the disease.

The application of multimodal imaging using fundus autofluorescence (FAF), optical coherence tomography (OCT), and OCT-angiography (OCTA) has profound implications for therapeutic targets, treatment strategies, and both clinical trial design and end-points.

Fundus autofluorescence (FAF) imaging of the retina has emerged as a useful, non-invasive imaging technique in diagnosis and follow-up of retinal dystrophies and in the monitoring of peripheral retina involvement. There are several different types of FAF, including blue-light autofluorescence (BAF), near-infrared autofluorescence (NIA), and short-wavelength (SW) FAF. BAF is the most used type of FAF and allows for the visualization of lipofuscin, the fluorescent pigment that accumulates in the RPE and photoreceptor cells, thus it is particularly useful for diagnosing macular dystrophies. FAF abnormalities depend on the examined macular dystrophies; however, late stages with RPE cell loss may look similar in various clinical entities. In this respect, FAF may be of limited use in the differential diagnosis. FAF was demonstrated to be useful in detecting retinal alterations before appearance of clinically visible abnormalities in both Best and Stargardt diseases [43]. Image acquisition can be difficult in patients with extensive macular dysfunction and/or atrophy who lack sufficient fixation.

OCT is an invaluable imaging modality in all macular diseases, as it allows the visualization of the retinal microarchitecture and thus a precise comprehension of ultrastructural pathological changes. OCT is enormously useful in diagnosis and clinical management of all different macular dystrophic entities. In most IMDs, changes take place at the level of the neurosensory retina and RPE. In Best VMD, OCT alterations have been demonstrated in all the progressive stages of the disease. In the previtelliform stage, the interdigitation zone may appear thicker and relatively hyperreflective. In subsequent stages, OCT best analyzes the subretinal vitelliform lesions anatomically; these lesions appear strongly hyperreflective and are located between the EZ and RPE [44]. In early-onset Stargardt disease, thickening of the external limiting membrane (ELM) can be used as a sign of early detection in the absence of other functional and structural retinal changes. In intermediate- and late-onset disease, OCT imaging reveals atrophy of the photoreceptor layers and of the RPE [45,46]. In X-linked Retinoschisis, macular schisis can be easily missed during clinical examination, making multimodal imaging invaluable. OCT can readily identify splitting of the inner and outer retinal layers. Cystic changes may be present in any layer of the retina and extend beyond visible fundus abnormalities [39]. In Pattern dystrophies changes are at the level of RPE, and in most cases display subretinal hyper-reflective material during OCT imaging [1]. Although inherited retinal diseases (IRDs) are not primarily vascular diseases, OCT-A is a useful method for studying vascular-related phenotypic aspects of macular dystrophies. Interestingly, retinal and choriocapillaris microvascular abnormalities have been reported in STGD patients when compared to controls. Mastropasqua et al. suggest the possibility of utilizing choriocapillaris dysfunction as a predictor for retinal function in STGD patients [47]. OCT-A is also implemented for detection of neovascular complications of macular dystrophies. In a prospective observational study published in Br J. Ophthalmology in 2018, OCT-A was proven to be superior to fluorescein angiography in detecting CNV in patients with Best disease because the vitelliform material masks CNV on FA, whereas OCT-A allows examination of vessels across different layers of the retina and choroid [37].

#### 2.2.2. Electrodiagnostic Tests (EDTS)

Despite the advent of the aforementioned sophisticated imaging methods, electrodiagnostic tests (EDTs) are still useful for the diagnosis of inherited macular dystrophies, especially in the differential diagnosis. Electrophysiological tests, such as full-field electroretinogram (ffERG), multifocal ERG (mfERG), Pattern ERG (PERG), and electroculogram (EOG), can be utilized to analyze and diagnose visual pathway malfunctions in macular dystrophies [48]. In Stargardt patients, given the significant heterogeneity of the condition, EDTs can help in the staging of the disease severity and in the monitoring of progression [31]. Best disease patients typically exhibit normal ffERG results, while mfERG often indicates reduced amplitudes in areas where subretinal fluids are present. The EOG, specific for this condition, is generally abnormal in symptomatic patients, and even in patients with a normal fundus presentation during the previtelliform stage [49]. Moreover, XLRS patients typically exhibit a classical electronegative response to a bright flash in a dark-adapted retina during an ffERG test [40].

## 3. Genetic Testing

The first issue to address is the evaluation of the appropriateness of a genetic test.

The identification of the genetic alteration(s) responsible for macular dystrophies, especially considering the extensive genetic heterogeneity, is required with the following scopes: to perform a correct gene-related definition of the condition for a possibly prognostic assessment, to identify pre-clinical affected family members for genetic counselling and reproductive issues, and to register patients into databases in order to potentially enroll them into present or future trials as retinal monogenic diseases are attractive targets for gene therapy [50,51]. Verma et al. proposed a protocol for genetic testing in ophthalmological diseases. The group highlighted the pivotal role of a careful family history and clinical evaluation in order to obtain enough data to support the hypothesis of the genetic origin of the disease before undertaking expensive and time-consuming genetic tests. The group proposed to perform chromosomal studies in cases with the presence of malformations, mental retardation, and/or dysmorphic features. In some instances, microarray studies should be undertaken, as these are able to identify subchromosomal anomalies such as micro-deletions [52]. Nevertheless, for most isolated retinal dystrophies and several syndromic conditions, intragenic variations as cause of the disease are the target of investigations. All aspects that can be related to genetic testing must be discussed with the patient in order to allow an informed choice.

If the pathological genetic condition is confirmed and the patient agrees, molecular testing is carried out.

In some cases, only a single gene is related to the condition, such as the *RS1* gene in X-linked retinoschisis; as such, direct Sanger Sequencing may be the first choice [53].

In most cases, the analysis of multiple potentially related genes must be conducted using a Next Generation Sequencing (NGS) technique, which can investigate, in parallel, a large amount of DNA. Strategies can differ, ranging from the analysis of a targeted panel of genes (TGS), the whole exome (WES), right up to whole genome sequencing (WGS). The wider the sequencing, the higher number of potential pathologic variants will be detected, with WGS having the potential to detect both exonic and intronic variants [54].

McClinton et al. recently described a strategy using single-molecule molecular inversion probe (smMIP)-based sequencing; this strategy provides a coverage of 97.4% of the *ABCA4* gene [55]. Despite the significant advantage of massive parallel sequencing, the main issue related to these new techniques is the consistent number of resulting information that requires a complex bioinformatic interpretation process and carries levels of uncertain significance [7].

To help interpret this massive amount of data, the American College of Medical Genetics and Genomics (ACMG) introduced a five-tier terminology system for identified variants after bioinformatic in silico evaluation: (1) benign, (2) likely benign, (3) uncertain significance (VUS), (4) likely pathogenic, or (5) pathogenic [56].

Establishing the effect of a variant can be challenging as current information is based on frequency in the population, public database, in silico prediction, and familial segregation of the variant being consistent with the affected/unaffected status. The clinical genetic report includes patient details and reason for referral, a result summary that discloses if there is a genetic variant consistent with the clinical suspect, further result information along with other variants of uncertain clinical significance [1], screened genes, and coverage reached.

When the clinical genetic report is received, in case of informative results (pathogenic and likely pathogenic variants consistent with the phenotype), the team should discuss with the patient the appropriateness of testing family members, as well as determining eligibility for clinical trials and eventually updating clinical management. In case of a type 3 result (uncertain clinical significance), testing of other family members can be carried out in order to verify segregation of the variant with the affected status. Family members genetic characterization is also necessary in recessive diseases to confirm compound double heterozygosity or even homozygosity. When in a recessive condition a single class 4 or 5 variant consistent with the phenotype is detected, copy number variants (CNVs) should be searched.

**Figure 5:**
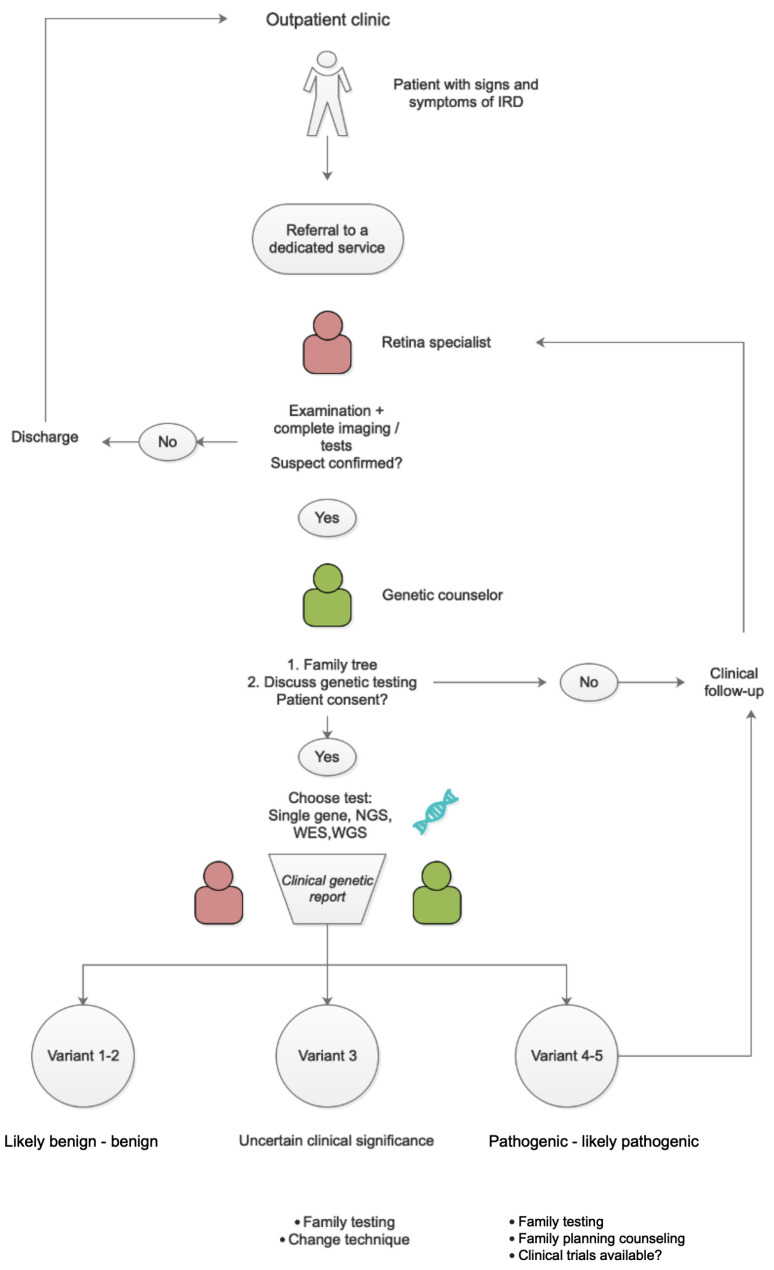
Flowchart with suggested patient pathway.

In case of type 2 and 1 (likely benign and benign) variants, the counselor can either confirm the absence of genetic disease or acknowledge the impossibility of identifying a genetic cause (Figure 5).

With next generation sequencing (NGS) technologies, panels of genes are evaluated at a reasonable cost in case multiple genes may be responsible for the clinical phenotype being investigated [53]. In case none of these gene results are informative, exome sequencing should be carried out. If this does not result in informative information, whole genome sequencing is a possibility.

## 4. Discussion

Most ophthalmologists are not familiar with genomic diagnostics; at present, this is a crucial tool in the management of IMDs and IRDs. Genetic testing has been found to be used in 1.5% of 207 patients with IRDs [57], with a great variability among different centers.

Genetic testing is indicated in a patient with a presumed diagnosis of IRD on the basis of clinical and instrumental findings, but it must not be used to exclude an IRD, as a negative result could reflect the limits of the analyzing technique (i.e., limited panel of genes) [58]. In some instances, no disease-related variants are found, possibly because the pathogenic defect lies in an unknown gene or in a region that is not covered by the sequencing technique.

Genetic testing is also used to assess the risk of recurrence in family members of affected patients. It is important to have a dedicated service for inherited retinal disease since this service will not only take care of the patient affected by the disease but it will also help to provide valuable information and support to family members who are at risk of developing the condition and are possibly in a pre-symptomatic phase. Once the pathogenic defect has been identified in the proband, its eventual presence can be traced in relatives and/or for reproductive issues. The careful integration of clinical, instrumental, and genetic data is crucial for the correct management of patients and their family members. Families affected or at risk for genetic disorders need genetic counsellors who can provide information and support during the investigations and the follow up. This is particularly important for *ABCA4*-related IMDs due to the considerable prevalence of heterozygous carriers in the general population, estimated to be between 1/20 and 1/50, depending on the different populations [59].

Genetic counsellors provide information about genetic testing, help families to understand the significance of genetic conditions and the spectrum of available therapeutic options, and can direct families to support groups and educational services. Due to the significant impact of genetic information, it is essential to thoroughly discuss all aspects of the results with patients. It is highly recommended to explain and document the discussion of these aspects, ensuring that patients fully understand the potential implications of genetic testing. This approach ensures that patients are aware of the benefits and risks of genetic testing, including the potential for incidental findings, as well as the limitations of genetic testing, such as false-positive and false-negative results. By discussing these aspects and documenting the discussion, patients can make informed decisions about whether to undergo genetic testing and, if so, what type of testing is appropriate for their situation. Overall, open and honest communication with patients regarding genetic testing is critical for providing quality care and optimizing patient outcomes. Genetic testing for patients with presumed or diagnosed inherited retinal disease is recommended by the American Academy of Ophthalmology Task Force on Genetic Testing and the European Reference Network for Rare Eye Diseases for all those conditions that have been related to a causative gene or genes [58,60].

Genotype-phenotype correlation can be very challenging in IMDs. Not only can the same phenotype be caused by different genes, but phenotypes caused by the same gene can be extremely variable. Even within a family, in a scenario described as “variable expressivity”, the same condition might present with different features. Another phenomenon that makes molecular diagnosis and familial segregation studies sometimes unclear is defined as “incomplete penetrance”. As previously described, individuals carrying a variant behaving as pathogenic in other family members may not display or complain of signs of the condition. These phenomena are thought to be caused by a range of different factors, including common variants, variants in regulatory regions, epigenetics, environmental factors, and lifestyle [61]. The extremely high number of interactions occurring in the visual cycle probably creates a particular milieu for accessory factors to modify the final outcome of genetically determined conditions.

The effects of genetic variants on the protein function can be different. In this respect, Variant Effect Predictors (VEPs) can provide useful information. Understanding the different behaviors of protein function deriving from DNA mutation can provide a useful tool to define the pathogenetic pathway and in the perspective of possible future therapies. DNA pathogenic variants can essentially cause different types of malfunctions. Loss of function variants (LOF) result in a gene product having less or no function; hypomorphic variants are the definition of those causing a partial loss of the normal activity of the protein. Gain of function (GOF) variants determine the production of a protein with a different and abnormal protein function, while dominant negative (DN) effect variants lead to the production of a protein that interferes with the activity of the wild-type [62]. The definition of the type of variant is extremely important in the final genotype-phenotype correlation. Unsurprisingly, we often encounter hypomorphic variants in the milder phenotypes. This is particularly true for *ABCA4*-related conditions, which are characterized by allelic heterogeneity, where the combination of different degrees of severity of the two variants (and the residual *ABCA4* protein function) influence the resulting phenotype [63].

In this context, there is a clear need for a dedicated service with defined roles. Based on current literature and our professional experience, we suggest the following scheme.

## 5. Therapeutic Options

Inherited retinal disease are vision-threatening disorders that lead to severe visual impairment and blindness; their management focuses on strategies to improve the use of residual vision and on genetic counselling. A comprehensive cover of therapeutic options is out of the scope of this review; therefore, techniques are briefly mentioned to inform the reader of this therapeutic possibilities.

### 5.1. Gene Therapy

Thanks to the monogenic nature of most of these diseases, the immunotolerant environment of the retina, and the improved molecular and genetic diagnosis, gene therapy has become a challenging new potential therapeutic strategy. Gene therapy uses genetic material (DNA or RNA) to modify gene expression; it can restore or inactivate a gene function on the basis of the type on mutation (loss or gain of function). The gene delivery approaches are currently based on the virus-mediated or the physical mechanism delivery of nanoparticles [64]. The prevalently used viral vectors are adenovirus (AV), lentivirus, and adeno-associated virus (AAV). AAV vectors are those most preferred for retinal disease as they are not pathogenic in humans in the wild-type, they have low immunogenicity and low retinal toxicity, and they do not integrate into the genome, even when providing efficient and long-term expression in retinal cells [65,66]. Gene therapy vectors can be introduced to the retina either with a subretinal injection or with intravitreal injections (with a much lower efficiency of transfection). Recently, suprachoroidal access is being investigated for a more efficient and safer access [67,68,69].

The first in vivo gene therapy, approved by FDA in December 2017, is voretigene neparvovec (Luxturna, Novartis Pharma GmbH). Luxturna is an adeno-associated virus vector carrying the *RPE65* gene for *RPE65*-associated retinal dystrophies [70]. Luxturna gene augmentation was shown to improve the visual function in *RPE65*-mediated LCA with a good safety profile [71]. Along with *RPE65*-mediated IRDs, other monogenic retinal dystrophies have been the focus of gene therapy studies, particularly for *ABCA4* [72] and *RS1*-related IMDs.

The *ABCA4* gene is particularly large in size; this presents a limitation in the use of AAV vectors, as these have a maximum capacity of accommodating 4.7 DNA kilobases. A dual AAV strategy, where the gene is administered through two separate vectors and subsequently re-assembles, has been successfully tested in animal models [32]. Lentiviral vectors have the advantage of a larger cargo capacity of about 8 kilobases, and are therefore an option for *ABCA4* gene delivery. Despite a limited expression at the photoreceptor level, results of these strategy have been encouraging in animal models [33] and clinical trials are currently undertaken. Although still at an experimental stage, non-viral delivery systems appear promising, especially due to the safer profile in terms of ocular and systemic toxicity, risk of insertional mutagenesis. and immune response [34].

There are six currently registered clinical trials involving the *RS1* gene, of which three are interventional [73].

### 5.2. Gene Editing

Gene editing techniques (Cas9) have shown promising results in correcting genetic mutations in a variety of genetic diseases, including cystic fibrosis, sickle cell anemia, and Huntington’s disease. In particular, clustered regularly interspaced short palindromic repeat (CRISPR)-associated protein (Cas) and an RNA that guides the Cas protein to a determined area of the genome is an appealing approach to challenge IRDs without available cure [74].

### 5.3. Optogenetics

Optogenetics is an emerging field that combines the use of genetic engineering and light to control the activity of specific cells in living organisms. As a therapeutic option, optogenetics shows great promise in treating genetic diseases by providing precise and targeted control. This can be attained by delivering genes encoding opsins, which are light sensitive transmembrane proteins, to be expressed ectopically in the target retinal cell [75].

### 5.4. Drug Therapies

Extensive research has been conducted on Docosahexaenoic Acid (DHA) and its potential therapeutic effects on retinal genetic diseases. As an omega-3 fatty acid, DHA plays a crucial role in the development and maintenance of the nervous system, including the retina. Studies conducted by Hoffman et al. indicate that DHA can slow the decline of field sensitivity in patients affected by X-linked retinitis pigmentosa [41]. 4-Methylpyrazole, a medication used to treat methanol poisoning, was proposed by Jurgensmeier et al. as a potential treatment for Stargardt disease. However, subsequent studies yielded discouraging results, and the medication is not currently used to treat retinal genetic disorders [76]. Metformin hydrochloride, a medication commonly used to treat diabetes, has also been studied for its potential neuroprotective effects in retinal diseases. Recent research demonstrates the drug’s ability to suppress retinal angiogenesis and inflammation in vitro and in vivo [77]. STG-001 is a therapy in development to treat inherited retinal diseases caused by mutations in the RPGR gene, currently being investigated for use in patients with Stargardt disease [78]. Emuxistat is a drug in development to treat Stargardt disease-associated macular atrophy, with limited but promising results [79]. Tinlarebant reduces retinol levels, subsequently reducing cytotoxic lipids in the retina, and is currently undergoing phase 3 trials to determine whether it can slow progression in adolescent patients [78]. ALK-001 is a chemically modified vitamin A being studied for the prevention and treatment of Stargardt disease, with encouraging preliminary results but a lack of phase 3 data [35]. The MADEOS study investigated the effects of omega-3 fatty acid supplementation on patients with AMD and Stargardt disease, reporting improved BCVA; however, the sample was small [36]. Saffron has also been studied for its potential neuroprotective effects in retinal diseases, with short-term supplementation showing no negative effects on central retina electroretinographic responses [80]. These treatments and supplements offer potential hope for the treatment of various retinal genetic diseases.

## 6. Conclusions

Recent progress in genetic research has unveiled the potential of a genomic approach to ophthalmic genetic conditions. Molecular genetic characterization of each patient is crucial for several reasons: a precise disease classification, evaluation of the risk of recurrence in the family along with related counselling, and finally to create a database for potential access to therapeutic options.

The interpretation of the effect of a variant is often a challenging task within molecular testing, making the establishment of the phenotypic consequences of underlying genetic variation a complex process that needs a close interaction between clinicians, molecular geneticists, bioinformatics, and medical geneticists.

A standardized protocol should be adopted by tertiary care centers in order to provide efficient care in the multidisciplinary management of patients, particularly in the perspective of the emerging genetic therapies.

## Figures and Tables

**Table 2:** Sample of clinical assessment spreadsheet.

Medical History				
Age at birth				
Connatal/perinatal infections				
Onset (age at first symptoms appearance/first diagnosis)				
Progression				
**Family History**				
	*Number, sex (M/F)*	*Affected (Y/N)*	*Age of onset*	*Progression*
Children				
Parents				
Brothers-sisters				
Nephews-nieces				
Maternal grandparents				
Maternal uncles/aunts				
Maternal cousins				
Paternal grandparents				
Paternal uncles/aunts				
Paternal cousins				
**Family Tree**				
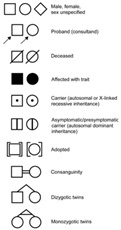				
**Inheritance Patterns Hints**				
Both sex equally affected?		Is there consanguinity?		→ *Autosomal recessive*
Both sex equally affected?		Are parents affected?		→ *Autosomal dominant*
Primarily affecting men?		No male-to-male transmission?		→ *X linked recessive*
Males with more severe symptoms?		No male-to-male transmission?		→ *X linked dominant*
Only maternal inheritance but both sexes affected?				→ *Mitochondrial*
Multiples affected siblings from healthy parents?				→ *Germline mosaicism*

## Data Availability

Data available upon request.

## References

[1] Rahman N., Georgiou M., Khan K.N., Michaelides M. (2020). Macular dystrophies: Clinical and imaging features, molecular genetics and therapeutic options. Br. J. Ophthalmol..

[2] Yla-Herttuala S. (2012). Gene therapy in age related macular degeneration and hereditary macular disorders. Front. Biosci..

[3] Baig A., Buckley D., Codina C. (2021). Behavioural adaptation to hereditary macular dystrophy: A systematic review on the effect of early onset central field loss on peripheral visual abilities. Br. Ir. Orthopt. J..

[4] Lois N., Holder G.E., Bunce C., Fitzke F.W., Bird A.C. (2001). Phenotypic subtypes of Stargardt macular dystrophy-fundus flavimaculatus. Arch. Ophthalmol..

[5] ABCA4 Gene. www.lovd.nl/ABCA4.

[6] Zernant J., Lee W., Nagasaki T., Collison F.T., Fishman G.A., Bertelsen M., Rosenberg T., Gouras P., Tsang S.H., Allikmets R. (2018). Extremely hypomorphic and severe deep intronic variants in the ABCA4 locus result in varying Stargardt disease phenotypes. Cold Spring Harb. Mol. Case Stud..

[7] Méjécase C., Malka S., Guan Z., Slater A., Arno G., Moosajee M. (2020). Practical guide to genetic screening for inherited eye diseases. Ther. Adv. Ophthalmol..

[8] Garg A., Lee W., Sengillo J.D., Allikmets R., Garg K., Tsang S.H. (2017). Peripapillary sparing in RDH12-associated Leber congenital amaurosis. Ophthalmic Genet..

[9] Amaral R.A.S., Zin O.A., Salles M.V., Motta F.L., Sallum J.M.F. (2023). Macular dystrophies associated with Stargardt-like phenotypes. Arq. Bras. Oftalmol..

[10] Dalvin L.A., Pulido J.S., Marmorstein A.D. (2017). Vitelliform dystrophies: Prevalence in Olmsted County, Minnesota, United States. Ophthalmic Genet..

[11] Johnson A.A., Guziewicz K.E., Lee C.J., Kalathur R.C., Pulido J.S., Marmorstein L.Y., Marmorstein A.D. (2017). Bestrophin 1 and retinal disease. Prog. Retin. Eye Res..

[12] Souied E.H., Querques G. (2016). Macular Dystrophies.

[13] Renner A.B., Fiebig B.S., Weber B.H.F., Wissinger B., Andreasson S., Gal A., Cropp E., Kohl S., Kellner U. (2009). Phenotypic variability and long-term follow-up of patients with known and novel PRPH2/RDS gene mutations. Am. J. Ophthalmol..

[14] Audo I., Sahel J., Mohand-Said S., Holder G., Moore A. (2016). X-Linked Retinoschisis. Macular Dystophies.

[15] Sikkink S.K., Biswas S., Parry N.R.A., Stanga P.E., Trump D. (2007). X-linked retinoschisis: An update. J. Med. Genet..

[16] Vijayasarathy C., Ziccardi L., Sieving P.A. (2012). Biology of retinoschisin. Adv. Exp. Med. Biol..

[17] Small K.W., DeLuca A.P., Whitmore S.S., Rosenberg T., Silva-Garcia R., Udar N., Puech B., Garcia C.A., Rice T.A., Fishman G.A. (2016). North Carolina Macular Dystrophy Is Caused by Dysregulation of the Retinal Transcription Factor PRDM13. Ophthalmology.

[18] Bowne S.J., Sullivan L.S., Wheaton D.K., Locke K.G., Jones K.D., Koboldt D.C., Fulton R.S., Wilson R.K., Blanton S.H., Birch D.G. (2016). North Carolina macular dystrophy (MCDR1) caused by a novel tandem duplication of the PRDM13 gene. Mol. Vis..

[19] Vaclavik V., Munier F. (2016). Malattia Leventinese (Autosomal Dominant Drusen). Macular Dystophies.

[20] Tsang S.H., Sharma T. (2018). Doyne Honeycomb Retinal Dystrophy (Malattia Leventinese, Autosomal Dominant Drusen). Adv. Exp. Med. Biol..

[21] Tsokolas G. (2022). Sorsby fundus dystrophy (SFD): A narrative review. Medicine.

[22] Davidson A.E., Sergouniotis P.I., Mackay D.S., Wright G.A., Waseem N.H., Michaelides M., Holder G.E., Robson A.G., Moore A.T., Plagnol V. (2013). RP1L1 Variants are Associated with a Spectrum of Inherited Retinal Diseases Including Retinitis Pigmentosa and Occult Macular Dystrophy. Hum. Mutat..

[23] Bianco L., Arrigo A., Antropoli A., Carrera P., Spiga I., Patricelli M.G., Bandello F., Battaglia Parodi M. (2022). Multimodal imaging evaluation of occult macular dystrophy associated with a novel RP1L1 variant. Am. J. Ophthalmol. Case Rep..

[24] Oliveira-Ferreira C., Leuzinger-Dias M., Tavares-Ferreira J., Silva S.E., Brandão E., Falcão-Reis F., Rocha-Sousa A. (2019). Hypotrichosis with juvenile macular dystrophy. Ophthalmic Genet..

[25] Puech B., De Laey J.J., Holder G.E. (2014). Inherited Chorioretinal Dystrophies.

[26] D’Esposito F., Cennamo G., de Crecchio G., Maltese P.E., Cecchin S., Bertelli M., Ziccardi L., Veneruso P.E., Magli A., Cennamo G. (2018). Multimodal Imaging in Autosomal Dominant Cone-Rod Dystrophy Caused by Novel CRX Variant. Ophthalmic Res..

[27] Marino V., Dal Cortivo G., Oppici E., Maltese P.E., D’Esposito F., Manara E., Ziccardi L., Falsini B., Magli A., Bertelli M. (2018). A novel p.(Glu111Val) missense mutation in GUCA1A associated with cone-rod dystrophy leads to impaired calcium sensing and perturbed second messenger homeostasis in photoreceptors. Hum. Mol. Gen..

[28] Hsu S.T., Ponugoti A., Deaner J.D., Vajzovic L. (2021). Update on Retinal Drug Toxicities. Curr. Ophthalmol. Rep..

[29] Retinal Information Network. https://web.sph.uth.edu/RetNet/.

[30] Forrester J.V., Dick A.D., McMenamin P.G., Roberts F., Pearlman E. (2021). The Eye, Basic Sciences in Practice.

[31] Fujinami K., Lois N., Davidson A.E., Mackey D.S., Hogg C.R., Stone E.M., Tsunoda K., Tsubota K., Bunce C., Robson A.G. (2013). A longitudinal study of stargardt disease: Clinical and electrophysiologic assessment, progression, and genotype correlations. Am. J. Ophthalmol..

[32] Trapani I., Boon C., Wijnholds J. (2018). Dual AAV Vectors for Stargardt Disease. Retinal Gene Therapy. Methods in Molecular Biology.

[33] Binley K., Widdowson P., Loader J., Kelleher M., Iqball S., Ferrige G., de Belin J., Carlucci M., Angell-Manning D., Hurst F. (2013). Transduction of photoreceptors with equine infectious anemia virus lentiviral vectors: Safety and biodistribution of StarGen for Stargardt disease. Investig. Ophthalmol. Vis. Sci..

[34] Huang D., Jeffery R.C.H., Aung-Htut M.T., McLenachan S., Fletcher S., Wilton S.D., Chen F.K. (2022). Stargardt disease and progress in therapeutic strategies. Ophthalmic Genet..

[35] Lu L.J., Liu J., Adelman R.A. (2017). Novel therapeutics for Stargardt disease. Graefe’s Arch. Clin. Exp. Ophthalmol..

[36] Prokopiou E., Kolovos P., Kalogerou M., Neokleous A., Nicolaou O., Sokratous K., Kyriacou K., Georgiou T. (2018). Omega-3 Fatty Acids Supplementation: Therapeutic Potential in a Mouse Model of Stargardt Disease. Investig. Ophthalmol. Vis. Sci..

[37] Guduru A., Gupta A., Tyagi M., Jalali S., Chhablani J. (2018). Optical coherence tomography angiography characterisation of Best disease and associated choroidal neovascularisation. Br. J. Ophthalmol..

[38] Michaelides M., Hunt D.M., Moore A.T. (2003). The genetics of inherited macular dystrophies. J. Med. Genet..

[39] Gerth C., Zawadzki R.J., Werner J.S., Héon E. (2008). Retinal morphological changes of patients with X-linked retinoschisis evaluated by Fourier-domain optical coherence tomography. Arch. Ophthalmol..

[40] Sieving P.A., Bingham E.L., Kemp J., Richards J., Hiriyanna K. (1999). Juvenile X-linked retinoschisis from XLRS1 Arg213Trp mutation with preservation of the electroretinogram scotopic b-wave. Am. J. Ophthalmol..

[41] Hoffman D.R., Hughbanks-Wheaton D.K., Spencer R., Fish G.E., Pearson N.S., Wang Y.-Z., Klein M., Takacs A., Locke K.G., Birch D.G. (2015). Docosahexaenoic Acid Slows Visual Field Progression in X-Linked Retinitis Pigmentosa: Ancillary Outcomes of the DHAX Trial. Investig. Ophthalmol. Vis. Sci..

[42] Chowers I., Boon C. (2016). The Pattern Dystrophies. Macular Dystophies.

[43] Boon C.J.F., Jeroen Klevering B., Keunen J.E.E., Hoyng C.B., Theelen T. (2008). Fundus autofluorescence imaging of retinal dystrophies. Vis. Res..

[44] Querques G., Regenbogen M., Quijano C., Delphin N., Soubrane G., Souied E.H. (2008). High-definition optical coherence tomography features in vitelliform macular dystrophy. Am. J. Ophthalmol..

[45] Nõupuu K., Lee W., Zernant J., Tsang S.H., Allikmets R. (2014). Structural and genetic assessment of the ABCA4-associated optical gap phenotype. Investig. Ophthalmol. Vis. Sci..

[46] Lee W., Nõupuu K., Oll M., Duncker T., Burke T., Zernant J., Bearelly S., Tsang S.T.H., Sparrow J.R., Allikmets R. (2014). The external limiting membrane in early-onset Stargardt disease. Investig. Ophthalmol. Vis. Sci..

[47] Mastropasqua R., Toto L., Borrelli E., Di Antonio L., Mattei P.A., Senatore A., Di Nicola M., Mariotti C. (2017). Optical Coherence Tomography Angiography Findings in Stargardt Disease. PLoS ONE.

[48] Chiang T.K., Yu M. (2023). Electrophysiological Evaluation of Macular Dystrophies. J. Clin. Med..

[49] Padhy S.K., Parameswarappa D.C., Agarwal K., Takkar B., Behera S., Panchal B., Ramappa M., Padhi T.R., Jalali S. (2022). Clinical and visual electrophysiological characteristics of vitelliform macular dystrophies in the first decade of life. Indian J. Ophthalmol..

[50] Stradiotto E., Allegrini D., Fossati G., Raimondi R., Sorrentino T., Tripepi D., Barone G., Inforzato A., Romano M.R. (2022). Genetic Aspects of Age-Related Macular Degeneration and Their Therapeutic Potential. Int. J. Mol. Sci..

[51] Hohman T.C. (2017). Hereditary Retinal Dystrophy. Handb. Exp. Pharmacol..

[52] Verma I.C., Paliwal P., Singh K. (2018). Genetic Testing in Pediatric Ophthalmology. Indian J. Pediatr..

[53] Tsang S.H., Sharma T. (2018). Genetic Testing for Inherited Retinal Dystrophy: Basic Understanding. Adv. Exp. Med. Biol..

[54] Khan M., Cornelis S.S., Pozo-Valero M.D., Whelan L., Runhart E.H., Mishra K., Bults F., AlSwaiti Y., AlTalbishi A., De Baere E. (2020). Resolving the dark matter of ABCA4 for 1054 Stargardt disease probands through integrated genomics and transcriptomics. Genet. Med..

[55] Mc Clinton B., Corradi Z., McKibbin M., Panneman D.M., Roosing S., Boonen E.G.M., Ali M., Watson C.M., Steel D.H., Cremers F.P.M. (2023). Effective smMIPs-Based Sequencing of Maculopathy-Associated Genes in Stargardt Disease Cases and Allied Maculopathies from the UK. Genes.

[56] Richards S., Aziz N., Bale S., Bick D., Das S., Gastier-Foster J., Grody W.W., Hedge M., Lyon E., Spector E. (2015). Standards and guidelines for the interpretation of sequence variants: A joint consensus recommendation of the American College of Medical Genetics and Genomics and the Association for Molecular Pathology. Genet. Med. Off. J. Am. Coll. Med. Genet..

[57] Neiweem A.E., Hariprasad S.M., Ciulla T.A. (2021). Genetic Testing Prevalence, Guidelines, and Pitfalls in Large, University-Based Medical Systems. Ophthalmic Surg. Lasers Imaging Retina.

[58] Stone E.M., Aldave A.J., Drack A.V., Maccumber M.W., Sheffield V.C., Traboulsi E., Weleber R.G. (2012). Recommendations for genetic testing of inherited eye diseases: Report of the American Academy of Ophthalmology task force on genetic testing. Ophthalmology.

[59] Al-Khuzaei S., Broadgate S., Foster C.R., Shah M., Yu J., Downes S.M., Halford S. (2021). An Overview of the Genetics of ABCA4 Retinopathies, an Evolving Story. Genes.

[60] Black G.C., Sergouniotis P., Sodi A., Leroy B.P., Van Cauwenbergh C., Liskova P., Grønskov K., Klett A., Kohl S., Taurina G. (2021). The need for widely available genomic testing in rare eye diseases: An ERN-EYE position statement. Orphanet. J. Rare Dis..

[61] Kingdom R., Wright C.F. (2022). Incomplete Penetrance and Variable Expressivity: From Clinical Studies to Population Cohorts. Front. Genet..

[62] Gerasimavicius L., Livesey B.J., Marsh J.A. (2022). Loss-of-function, gain-of-function and dominant-negative mutations have profoundly different effects on protein structure. Nat Commun..

[63] Cevik S., Biswas S.B., Biswas-Fiss E.E. (2023). Structural and Pathogenic Impacts of ABCA4 Variants in Retinal Degenerations-An In-Silico Study. Int. J. Mol. Sci..

[64] Chiu W., Lin T.-Y., Chang Y.-C., Lai H.I.-A.M., Lin S.-C., Ma C., Yarmishyn A.A., Lin S.-C., Chang K.-J., Chou Y.-B. (2021). An Update on Gene Therapy for Inherited Retinal Dystrophy: Experience in Leber Congenital Amaurosis Clinical Trials. Int. J. Mol. Sci..

[65] Surace E.M., Auricchio A. (2008). Versatility of AAV vectors for retinal gene transfer. Vis. Res..

[66] Moore N.A., Morral N., Ciulla T.A., Bracha P. (2018). Gene therapy for inherited retinal and optic nerve degenerations. Expert Opin. Biol. Ther..

[67] Ochakovski G.A., Bartz-Schmidt K.U., Fischer M.D. (2017). Retinal Gene Therapy: Surgical Vector Delivery in the Translation to Clinical Trials. Front. Neurosci..

[68] Davis J.L., Gregori N.Z., MacLaren R.E., Lam B.L. (2019). Surgical Technique for Subretinal Gene Therapy in Humans with Inherited Retinal Degeneration. Retina.

[69] Scherbakova I., Ragi S.D., Sharma T. (2023). Ocular Injection Techniques for Retinitis Pigmentosa: Intravitreal, Subretinal, and Suprachoroidal. Methods Mol. Biol..

[70] U.S. Food and Drug Administration (2017). Voretigene Neparvovec-rzyl BL125610/0. https://www.fda.gov/media/109487/download.

[71] Russell S., Bennett J., Wellman J.A., Chung D.C., Yu Z.-F., Tillman A., Wittes J., Pappas J., Elci O., McCague S. (2017). Efficacy and safety of voretigene neparvovec (AAV2-hRPE65v2) in patients with RPE65-mediated inherited retinal dystrophy: A randomised, controlled, open-label, phase 3 trial. Lancet.

[72] Tamboli V., Mishra G.P., Mitrat A.K. (2011). Polymeric vectors for ocular gene delivery. Ther. Deliv..

[73] Clinicaltrials.gov Webpage. clinicaltrials.gov.

[74] Benati D., Patrizi C., Recchia A. (2020). Gene editing prospects for treating inherited retinal diseases. J. Med. Genet..

[75] John M.C., Quinn J., Hu M.L., Cehajic-Kapetanovic J., Xue K. (2023). Gene-agnostic therapeutic approaches for inherited retinal degenerations. Front. Mol. Neurosci..

[76] Jurgensmeier C., Bhosale P., Bernstein P.S. (2007). Evaluation of 4-methylpyrazole as a potential therapeutic dark adaptation inhibitor. Curr. Eye Res..

[77] Han J., Li Y., Liu X., Zhou T., Sun H., Edwards P., Gao H., Yu F.-S., Qiao X. (2018). Metformin suppresses retinal angiogenesis and inflammation in vitro and in vivo. PLoS ONE.

[78] Kim N., Priefer R. (2021). Retinol binding protein 4 antagonists and protein synthesis inhibitors: Potential for therapeutic development. Eur. J. Med. Chem..

[79] Kubota R., Birch D.G., Gregory J.K., Koester J.M. (2022). Randomised study evaluating the pharmacodynamics of emixustat hydrochloride in subjects with macular atrophy secondary to Stargardt disease. Br. J. Ophthalmol..

[80] Piccardi M., Fadda A., Martelli F., Marangoni D., Magli A., Minnella A.M., Bertelli M., Di Marco S., Bisti S., Falsini B. (2019). Antioxidant Saffron and Central Retinal Function in ABCA4-Related Stargardt Macular Dystrophy. Nutrients.

